# Mapping the Diversity of Maize Races in Mexico

**DOI:** 10.1371/journal.pone.0114657

**Published:** 2014-12-08

**Authors:** Hugo Perales, Duncan Golicher

**Affiliations:** 1 El Colegio de la Frontera Sur, Grupo de Agroecología, Carretera Panamericana y Periférico Sur s/n, San Cristóbal, Chiapas, Mexico; 2 El Colegio de la Frontera Sur, Grupo Conservación y Restauración de Bosques, Carretera Panamericana y Periférico Sur s/n, San Cristóbal, Chiapas, Mexico; 3 Center for Conservation Ecology & Environmental Change, School of Applied Sciences, Bournemouth University, Dorset, United Kingdom; Cairo University, Egypt

## Abstract

Traditional landraces of maize are cultivated throughout more than one-half of Mexico's cropland. Efforts to organize *in situ* conservation of this important genetic resource have been limited by the lack of knowledge of regional diversity patterns. We used recent and historic collections of maize classified for race type to determine biogeographic regions and centers of landrace diversity. We also analyzed how diversity has changed over the last sixty years. Based on racial composition of maize we found that Mexico can be divided into 11 biogeographic regions. Six of these biogeographic regions are in the center and west of the country and contain more than 90% of the reported samples for 38 of the 47 races studied; these six regions are also the most diverse. We found no evidence of rapid overall decline in landrace diversity for this period. However, several races are now less frequently reported and two regions seem to support lower diversity than in previous collection periods. Our results are consistent with a previous hypothesis for diversification centers and for migration routes of original maize populations merging in western central Mexico. We provide maps of regional diversity patterns and landrace based biogeographic regions that may guide efforts to conserve maize genetic resources.

## Introduction

Extant diversity of native landraces of maize (*Zea mays* L. subsp. *mays*) in Mexico is abundant and has attracted scholars interested in the use and the understanding of the production and patterns of maize diversity. Selection both by farmers and by environmental factors led to the evolution of a large number of distinct landraces. Continuous variation, convergent morphological evolution, plasticity with respect to environmental conditions, and the rich traditional culture associated with the crop [1] all add to the challenges involved in unraveling the complex spatial pattern of maize diversity in Mexico.

Global maize production exceeds that of all other cereals [2]. The crop is particularly important in Mexico and Central America, where it provides the staple food. Maize was first domesticated 6,000 to 10,000 years ago in South West Mexico (3, 4) and is possibly the most diverse crop species known [5]. Variability within Mexico is exceptionally high, where maize is cultivated in a wide range of environments from sea level to more than 3000 masl and from tropical humid environments to semi-desert conditions. More than 2.5 million Mexican farmers plant about 8 million hectares annually [6] with over 75% of the seed that is sown saved by farmers from their previous harvest [7]. Landraces comprise at least one-half of the seed planted each year in Mexico.

Studies of the biogeography of crops often focus on determining the region or center of origin and domestication of the species [8], [9]. Interest in using genetic resources from the centers of domestication for breeding purposes has motivated these studies [9], [10]. The history of maize domestication has been intensively studied and has progressively focused on specifying the probable area where domestication first took place. Starting from a general American cradle, “possibly in Colombia” [8], it went from an explicit Mesoamerica origin [9] to present expectations of a single domestication center in the basin of the Balsas River drainage where Michoacan, Guerrero and the State of Mexico meet [2], [3]. An alternative multiple domestication model based on chromosomal characteristics [11], [12] proposes five domestication centers and four diversification centers.

Interest in using maize genetic resources led to the creation of a system for racial classification of maize diversity. Racial classification of maize was originally proposed by Anderson and Cutler [13] and formalized by Wellhausen et al. [14]. Dissatisfied with the previous artificial classification of maize based on the composition of the endosperm of the kernel [15], Anderson and Cutler [13] aimed for a natural classification. They proposed a racial classification system that could reflect the history and relationships of the constituent groups. Aware of the continuous variation in many maize characters, they defined race “as loosely as possible” as “a group of related individuals with enough characteristics in common to permit their recognition as a group”, expecting that a race had “a significant number of genes in common, major races having a smaller number in common than do sub-races” [13]. Anderson and Cutler [13] emphasized that the analysis of races was primarily of groups and not of separate individuals. The racial system for maize classification has also been used in other countries [16] and remains an important benchmark for sampling diversity in genetic studies [3], [17], [18], [19]. The terms landrace and race are not interchangeable for maize in Mexico. A landrace is a crop population with historical origin, distinct identity and that lacks formal crop improvement; it is often genetically diverse, locally adapted and associated with traditional farming systems [20]. As described, a maize race seeks to be a natural classification system although it is not a formal taxonomic unit for crops [21]. In Mexico all landraces can be classified in a race category, while some races have both landrace and formal commercial cultivars.

After an extensive sample of Mexican maize between 1943 and 1951, Wellhausen et al. [14] created the basis of the widely accepted classification system we use presently. Wellhausen et al. [14] based their groupings on morphological, genetic, cytological, physiological, and agronomic characteristics and gave special consideration to geographic distribution, which was considered of great importance in recognizing races. They described the distribution for each race and mapped the collection points of the sample. Notwithstanding the recognized importance of the geographic distribution, very little work has been done to advance the subject in the last 60 years (for an exception see [22]).

It should be noted that morphological diversity of maize and its classification does not correspond directly to neutral genetic diversity, at least not for a few markers. Pressoir and Berthaud [23], [24] and van Heerwaarden et al. [25] found relatively low between population genetic differentiation for landraces and races while observing strong divergent selection for flowering precocity [23] and ear morphological characters [24] or for both vegetative and ear morphology [25]. Vigouroux *et al*. [19] reported a low correlation between race name and genetic distance. Nonetheless, farmers organize variety management based on “types” [26], for example, including all hybrids as one type but also distinguishing particular landraces in a similar fashion to racial classification.

At the beginning of the exploratory work to classify maize Anderson [27] noted that “maize is a sensitive mirror of the people who grow it”. We expect that the distribution of maize races is determined by environmental [28] and also by social and cultural factors [1]. For the case of Mexico, a possible relationship between indigenous groups and maize diversity has been proposed [1] and recently tested [29], [30]. Although it seems that climate could be the strongest driver that explains maize distribution [30], we can suppose that the extensive history of the more than 60 indigenous groups extant in Mexico have also left their mark.

Even though maize has been the most studied crop in Mexico, we lack a national perspective of spatial diversity patterns to identify priority areas and organize *in situ* conservation efforts for maize genetic resources. In 2005 the Mexican Government began an ambitious research program, coordinated by CONABIO (Comisión Nacional para el Conocimiento y Uso de la Biodiversidad), aimed at surveying the current diversity of maize races throughout the national territory. Here we use the dataset from this project to investigate biogeographic patterns and centers of diversity for the Mexican races and how these centers have changed over the previous sixty years. The dataset analyzed consists of 18,439 geo-referenced collections with racial classification dating from 1934 to 2010. Models were created by GAM (general additive models) using interpolated climate surfaces for the data set segmented by collection effort for three time periods of about 10 years around 1950, 1975 and 2005, and for the whole data set (see method for details).

In this report we will show that maize diversity is not evenly distributed throughout Mexico and propose six diversity centers. Based on the spatial analysis of racial composition we also pose 11 biogeographic regions, six of which correspond closely with the six diversity centers. Our analysis of the three collection efforts indicate that maize diversity has remained relatively stable since formal collections began more than 60 years ago. Finally, we will suggest that explaining the distribution of maize also requires looking at socioeconomic factors and the distribution of cultural diversity in Mexico, though no straightforward relationship is evident.

## Results

Even though there are 59 reputed races identified for Mexico [17], [31], we worked with only 47 races because 9 had very small samples (<15 samples, S1 Table) to allow their spatial distribution to be mapped with some confidence, two were synonyms and one is not considered Mexican. Relative frequency of the 47 races studied did not change dramatically between collection efforts, even though sampling biases are extensive. Collector's criteria for sampling have not been consistent and have depended on individual criteria. Spearman's rank correlation for race frequency between the three collection efforts was highly significant for all cases (p<0.0001; Rho for 1950*1975 = 0.72, 1950*2005 = 0.63, and 1975*2005 = 0.73) and also between each of these efforts and the whole sample (p<0.0001; Rho 0.77, 0.86, and 0.95, respectively for 1950, 1975 and 2005). Throughout the 60 years of collection five races were consistently very common within collection effort and twenty races were rare (<100 samples since 1943), but none of the rare races had been notably more abundant in historical records than in recent collections. Several of these races were described as very rare since the 1940's [13]. Two very common races (Tuxpeño and Celaya) seem to have increased in frequency and distribution.

**Figure 1:**
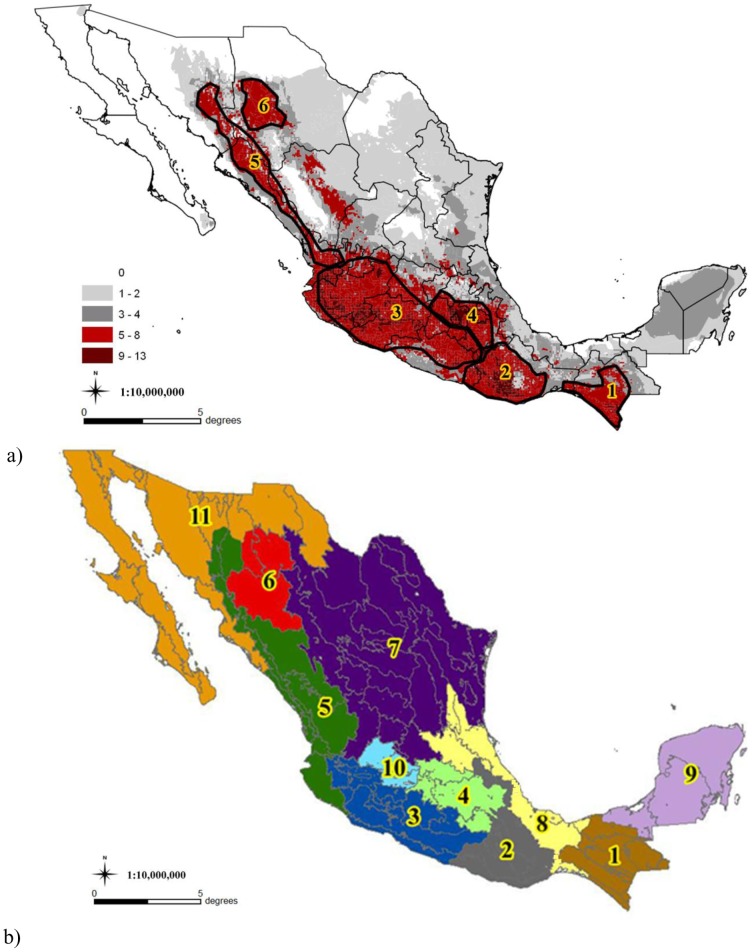
Diversity areas and biogeographic regions for the races of maize in Mexico. a) Race richness (number of races occurring within each grid cell) and diversity centers and b) biogeographic regions for the races of maize in Mexico (see also S2 Figure). Six biogeographic regions are also diversity centers (numbers 1 to 6): 1) Chiapas Complex, 2) Oaxacan Valleys and Sierras, 3) Western Costal Mountain Range, 4) Central Plateau, 5) Northwest Sierras, 6) Chihuahuan Canyons, 7) Northern Plateau, 8) Gulf and Isthmus Plains, 9) Yucatán Peninsula, 10) Bajío, and 11) Baja California and Northwest.

Our results indicate the existence of 11 biogeographic regions, six of which contain high diversity areas (Fig. 1, regions 1 to 6). The indication of the six diversity regions was apparent from qualitative inspection of the composite distribution maps for each collection effort (S2 Figure), though we were able to formalize the areas based on the regional clusters formed through spatial analysis of racial composition (S3 Figure). These six regions are in correspondence both for high race richness (i.e. number of races) and distinct racial composition, each region contains a large proportion of the distribution of several races which distinguished it with respect to the other biogeographic regions for maize (Table 1). Taken together the six high diversity regions contain 38 of the 47 races (80.9%) we studied and hold more than 90% of all the samples for these 38 races.

**Table 1:** Maize biogeographic regions for Mexico, associated races and proportion of all collections of the race within the region between 1943 and 2010.

Region number	Region name	Name in Spanish	Characteristic races		Other races with significant presence	
			Name	Collections of race (%)	Name	Collections of race (%)
1	Chiapas Complex	Complejo de Chiapas	Comiteco	95.7	Olotillo	19.2
			Olotón	78.6	Tepecintle	15.0
			Tehua	95.3	Tuxpeño	33.4
					Vandeño	20.2
					Zapalote Grande	38.6
2	Oaxacan Valleys and Sierras	Valles y Sierras de Oaxaca	Bolita	79.1	Nal Tel	24.2
			Chiquito	87.1	Olotillo	32.8
			Coscomatepec	66.1	Olotón	16.6
			Mushito	44.7	Zapalote Chico	25.4
			Serrano Mixe	97.4	Zapalote Grande	28.9
			Tepecintle	58.5		
3	Western Costal Mountain Range	Cordillera Costera de Occidente	Ancho	38.3	Celaya	17.5
			Bofo	49.9	Jala	15.0
			Complejo Serrano de Jalisco	88.9	Maíz Dulce	16.4
			Conejo	66.7	Reventador	35.7
			Elotero de Sinaloa	52.1	Tabloncillo	27.2
			Mushito	40.1	Tabloncillo Perla	16.7
			Pepitilla	61.6		
			Vandeño	40.4		
			Zamorano Amarillo	62.3		
4	Central Plateau	Mesa Central	Ancho	51.4	Coscomatepec	21.2
			Arrocillo Amarillo	55.0	Maíz Dulce	20.0
			Cacahuacintle	93.8	Pepitilla	25.2
			Chalqueño	75.2		
			Cónico	97.6		
			Elotes Cónicos	70.9		
			Palomero Toluqueño	90.0		
5	Northwest Sierras	Sierras del Noroeste	Chapalote	100.0	Maíz Dulce	16.4
			Dulcillo del Noroeste	88.5	Vandeño	11.1
			Elotero de Sinaloa	47.9		
			Jala	85.0		
			Maíz Blando de Sonora	100.0		
			Onaveño	78.5		
			Reventador	60.7		
			Tablilla de Ocho	45.2		
			Tabloncillo	54.8		
			Tabloncillo Perla	81.9		
6	Chihuahuan Canyons	Cañones Chihuahuenses	Apachito	83.1	Tablilla de Ocho	16.7
			Azul	93.5		
			Cristalino de Chihuahua	90.3		
			Gordo	66.7		
7	Northern Plateau	Mesa del Norte	Cónico Norteño	70.2	Celaya	18.4
			Ratón	71.6	Gordo	17.4
			Tuxpeño Norteño	82.2	Tablilla de Ocho	26.2
8	Gulf and Isthmus Plains	Planicies del Golfo e Istmo	Olotillo	38.6	Arrocillo Amarillo	16.7
			Tuxpeño	19.7	Ratón	18.1
			Zapalote Chico	61.6	Zapalote Grande	18.1
9	Yucatan Peninsula	Península de Yucatán	Dzit-Bacal	86.9	Tuxpeño	19.7
			Nal-Tel	65.9		
10	Bajio	Bajío	Celaya	35.9	Zamorano Amarillo	24.7
			Maíz Dulce	30.9		
11	Baja California and Northwest	Baja California y Noroeste	none		none	

In the south of Mexico the Chiapas Complex region corresponds almost completely to the state of Chiapas and has nearly all the collections of the races Tehua and Comiteco and most of Oloton. The last two races are extensively shared with Guatemala [32] but only slightly with Oaxaca and other states of Mexico. The Oaxacan Valleys and Sierras include the state of Oaxaca and also the southeastern side of Guerrero, eastern Puebla and adjacent highland areas of Veracruz. Distinctive races include Bolita, Nal-Tel del Altura (also known as Chiquito) and Serrano Mixe. The Western Costal Mountain Range includes most of the states of Guerrero, Michoacan and central Jalisco and has 9 distinctive races, though the collections of these races are shared with neighboring regions. The Central Plateau comprises nearly all of the state of Mexico and Puebla, all of Tlaxcala, Distrito Federal and some areas of Michoacán, Queretaro and Hidalgo. Distinctive races of the Central Plateau are Conico, Cacahuacintle and Palomero Toluqueño with large proportions of the collections of Chalqueño and Elotes Conicos. The Northwestern Sierras comprises western and north Jalisco, all of Nayarit, eastern Sinaloa and western Durango and the southeast of Sonora. The area with the most diversity is at the foothills on the west side of the mountain range, the race Jala is almost endemic to this region although 10 other races have significant proportions of collections that are shared with the south of the Baja California and the Northwest region, notably Chapalote, Dulcillo del Noroeste, Maiz Blando de Sonora, Onaveño, and Reventador. Chihuahuan Canyons is contained within the southwest of Chihuahua and almost all collections of Apachito, Azul, Cristalino de Chihuahua and Gordo are in this region.

Five other biogeographic regions can also be characterized but these do not hold high diversity, with one exception. Bajio is a small region in southern Guanajuato, northeast Jalisco and Michoacan with high diversity (Table 2) but it shares racial composition with the Western Coastal Mountain Range, the Central Plateau and the Northern Plateau. None of its distinctive races (Table 1) are endemic to the region, with the possible exception of Celaya, and all are comparatively common in other regions. The high diversity of the Bajio seems to arise because the distribution models for several races include the region, though based on actual collections most of these races are in very low frequencies. For these reasons we do not advocate the Bajío as a seventh high diversity region, as a diversity region it can be subsumed within the Western Coastal Mountain Range. Two other biogeographic regions that have relative low diversity are characterized by races that are almost endemic to the region. The Northern Plateau is the largest region comprising several states of northeastern and north central Mexico. This region has almost all the collections of Conico Norteño, Raton and Tuxpeño Norteño. The Yucatan Peninsula comprises the states of Yucatan, Quintana Roo and Campeche and has almost all collections of the Dzit-Bacal race. The Gulf and Isthmus Plains is comprised mostly by the state of Veracruz, but also includes eastern San Luis Potosi, Queretaro and Hidalgo and the isthmus region of Oaxaca. Although most of the races present in this region have low proportion of the collections of these races, the exception being Zapalote Chico in the isthmus, it is also the reputed origin of the Tuxpeño race, maybe the most important parental material in scientifically bred varieties for Mexico [33]. The region of the Baja California and Northwest has scarce agriculture and the lowest richness, even though in its southern side there are a few samples of races it shares with the Northwestern Sierras region.

**Table 2:** Mean richness (number of races occurring within each grid cell) for models of collection efforts by biogeographic region.

Biogeographic region	Number of grid cells		Models by collection effort				All data
	Total	%	1950	1975	2005	mean of 3 models	
n (accessions)			1,878	3,568	11,151		18,344
1. Chiapas Complex	1,666	3.8	2.90	3.94	3.11	3.32	4.35
2. Oaxacan Valleys and Sierras	1,994	4.6	3.75	4.05	4.60	4.13	5.88
3. Western Costal Mountain Range	3,157	7.2	4.47	3.10	5.67	4.42	6.63
4. Central Plateau	1,654	3.8	4.00	4.38	4.58	4.32	6.23
5. Northwest Sierras	4,227	9.7	0.99	3.75	3.10	2.61	4.22
6. Chihuahuan Canyons	1,476	3.4	0.53	3.43	3.94	2.64	4.08
7. Northern Plateau	13,258	30.3	1.30	1.37	1.63	1.44	1.86
8. Gulf and Isthmus Plains	2,478	5.7	2.33	2.03	2.67	2.35	2.96
9. Yucatan Peninsula	3,584	8.2	1.11	1.98	1.45	1.51	2.12
10. Bajio	748	1.7	6.58	7.15	4.29	6.01	6.47
11. Baja California and Northwest	9,551	21.8	0.06	0.73	0.40	0.40	0.53
Mean for Mexico			1.61	2.17	3.11	3.32	4.35

Comparing race richness for the models of the three collection efforts studied (1950, 1975, 2005) we did not find evidence of other diversity areas (S2 Figure), the positions of the centers of diversity have remained fairly constant with differences between collection efforts. Most richness differences between collection efforts can be attributed to variable sampling distribution and intensity. For example, for 1950 models Chiapas and the north of Mexico were sparsely collected or not at all and the diversity of these regions is lower than for 1975 and 2005 collection efforts. The Central Highlands and the west of Mexico (regions 1 to 6 in Fig. 1B) are the regions with the greatest diversity of maize races. The pattern of diversity was not affected when our models included the additional 10 races with small samples (<12) and 6 presumed new races not formally described that were left out in our main models.

We found scant evidence of an overall reduction in maize diversity when comparing richness over more than 50 years. Mean richness for grid cells for all Mexico was 1.61, 2.17 and 2.26 for 1950, 1975 and 2005 collection efforts, respectively (Table 2), and maximum richness was 12 for 1975 and 2005 and 10 for 1950. Lower richness for the 1950 models can be attributed to a smaller sample, fewer sites sampled and fewer recognized races; several races were described after 1970. Maximum richness for collection data without modelling was slightly lower and followed the same pattern. Richness by biogeographic region also has the same general pattern, with higher richness in the 2005 models than those for 1975 and 1950. At the regional level the Northwestern Sierras, the Chiapas Complex and the Yucatan Peninsula regions seem to show a trend to less diversity in 2005 models than in the two previous modeling periods (Table 2). This may reflect the increase in commercial production in Sinaloa and lowland Chiapas. Overall richness for Yucatán is low and the lower value for 2005 may reflect the decreased importance of Nal-Tel and Dzit-Bacal and increased dominance of Tuxpeño in the Peninsula.

All the 47 races we studied were sampled in the recent collection effort and most of these seem to have stable distributions throughout the 60-year period. Nonetheless, for 5 races with limited distributions (Chiquito, Jala, Olotón, Palomero Toluqueño and Zapalote Chico) our models suggest a minor decline in their distribution, though in the case of Oloton it might be due to sampling differences. Also, six races (Chapalote, Complejo Serrano de Jalisco, Dulcillo del Noroeste, Jala, Onaveño, Tablilla de Ocho) have dropped in reporting rate to the point that they can be considered at high risk of extinction (<15 samples in 11,151 accessions for the time period between 1997-2010). Additionally, it should be recalled that 7 formally described races have less than 15 samples in 60 years of collection efforts (not included in our main models, see methods). Though we didn't find evidence of race extinction or significant decline in distributions, several races may be vulnerable.

## Discussion

### Changes in race abundance, distribution and richness

Estimation of changes in race abundance based on their frequency in collections or distribution models are always tentative. There are differences in regional collection efforts and sampling criteria of the more than 200 collectors involved since 1943 and models represent potential and not actual distributions. Nonetheless, several patterns are apparent.

The high rank correlation between the three collection efforts indicate that relative abundance of races has not changed considerably since 1950. Races that were common in 1950 were also common in 2005, and the same can be said for rare races, none of these were apparently more common in 1950. Correspondingly, the comparison of distribution and richness models for the three sampling efforts does not suggest a general decline in richness nor in distribution for 43 of the 47 races studied, and in the other four cases the declines are relatively minor and require verification. The recent increase in distribution for two races (Tuxpeño and Celaya) may be explained by the outstanding importance these have had as components in commercial cultivars [33], it is not uncommon to find recycled and creolized landraces that had their origin as commercial seeds [34]. Also, no extinctions were reported for the 47 races studied since all were sampled in the last collection effort between 1997 and 2010. Therefore, in a broad sense, at the race level of classification we do not find signs of substantial genetic erosion between 1943 and 2010. That is not to say no losses of local populations have occurred.

Even though no major genetic erosion appears to have occurred, 7 races had very small frequencies in recent collections, 13 races were very rare and 10 formally described races were not included in this study because of very small samples. That is, although these races might have been rare for the last 60 years, half of the 59 formal races cannot be considered to have stable enough conditions for long term *in situ* conservation. The latter is particularly significant if we consider possible distribution decline under climate change scenarios where highland races [35] and races that have low abundance and limited distribution may be particularly vulnerable [36]. Also, three of the eleven biogeographic regions proposed for maize suggest a decrease in diversity, possibly due to the use of commercial cultivars. The Northwestern Sierras region is adjacent to very intensively managed maize in Sinaloa, and in Chiapas the areas that show a decrease in diversity have relatively intensive maize production with important use of commercial cultivars. In Yucatán the decrease could also be linked to commercial cultivars but it might also be derived from the decreased importance of Nal-Tel and Dzit-Bacal races, which has been reported elsewhere [37].

We should note that the status of races with very small samples is suspect. Ten races have been described formally yet it seems that practically only the initial collections used some of these names. A reason for this may be due to the lack of a recent systematic publication that includes all races, descriptions after Wellhausen's et al. [14] are dispersed in an article, two Ph.D. dissertations [38], [39], [40], and an unpublished document. Also, it is always possible to find and classify off-type populations that later are not common in recurrent sampling efforts. Wellhausen et al. [14] noted that races are not “pure” and not all maize found could be assigned to one of the recognized races, many are mixtures of two or more. Anderson and Cutler [13] defined race for a group of related populations recognizable as a group, that is, a metapopulation with subdivided populations [41]. If an alleged race does not consist of a set of subdivided populations with significant territorial presence its existence can be called into question because of its incidental abundance. Nonetheless, the race category for classifying maize diversity seems robust and the distribution models for the races are consistent between three collection efforts, even though many collectors have been involved in the classification of the collections. In the case of Mexico the race classification for maize has been especially useful to sort out maize diversity at the national level.

### Diversity regions and Kato's centers of diversity

We found six high diversity centers based on race richness and composition. These six diversity centers agree well with Kato's [11], [12] proposed centers of diversification and his suggested consequences of possible migration routes of the original maize germplasm. Based on chromosome knob morphology Kato advocated that maize had a multicenter origin in four regions in Mexico and one in Guatemala, he also proposed four diversification centers for Mexico. Even though Kato's proposition of a multicenter origin for maize has not received additional support, and present evidence points to one center of origin [3], his depiction of diversification regions is useful for suggesting specific ancestral areas within Mexico.

The four diversification centers proposed by Kato [11], [12] correspond directly to our diversity regions. Two of Kato's diversification centers are essentially the same regions of our Central Plateau and Western Mountain Costal Range regions. We propose that the other two diversification centers should be divided based on racial composition (Fig. 1B and Table 1). Kato's northwestern center includes our Chihuahuan Canyons and Northwestern Sierras maize diversity regions, which do not share their characteristic races (Table 1). The southeastern center of Kato corresponds to our Valleys and Sierras of Oaxaca and Chiapas Complex regions which, although sharing several races, contrast strongly in their composition (Table 1).

Race diversity is clearly higher in western and central Mexico than in the east and the north. Kato [12] also suggested migration routes of the original germplasm and noted that it was in western central Mexico that three routes merged, thus promoting greater diversity. A migration route going through the costal lands in western Mexico and by the highlands of northwestern Chihuahua into the United States was previously proposed (38, 42). In our analysis two of the regions with highest racial composition were the Western Mountain Costal Range, and the Valleys and Sierras of Oaxaca, both in western Mexico in the area suggested by Kato [12]. This greater diversity is also discernible based on the number of racial groups proposed by Sanchez *et al*. [17], [43] based on isozymes, morphological characters and genotype x environmental interaction. The Western Mountain Costal Range and the Valleys and Sierras of Oaxaca both presented three of the major race groups proposed by Sanchez *et al*. [17], [43], whereas in the other regions only one or two of the major groups were found. Thus, our results are consistent both with Kato's hypothesis for diversification centers and for migration routes of original maize populations merging on western central Mexico.

### Environment, social drivers, indigenous people and racial diversity of maize

We expect that maize race composition and diversity mirrors, to some extent, environmental structure and diversity [14], [28], but we also expect a relationship with cultural diversity and other social differentiation factors [27], [29], [30], [44], [45], [46], [47]. In the case of maize in Mexico the relationship between environmental and social drivers and diversity is not simple and there seems to be cases for both environmental and social driven signals.

It should be noted that the dataset under study is biased towards sampling only landraces and excluding hybrids and other commercial cultivars, this can produce spurious correlations when comparing race distributions with environmental and social factors. Nonetheless, we know that lands suitable for mechanization and irrigation, in particular in the north of Mexico, are correlated with the use of commercial cultivars and landraces are almost completely dominant in hillside agriculture and in the highlands [46]. In the present study we will not develop further socioeconomic factors because data for many relevant variables are available only at an aggregated scale (municipality) too coarse to be useful for seeking patterns. For social variables we will only consider the hypothesis that expects correlation of maize diversity with indigenous populations for which detailed population data is available.

It is well known that maize races and cultivars have a strong correlation with temperature and rainfall [14], [28], [30], [48]. For Mexico, there are three macro-environmental conditions for maize based largely on temperature. Ruiz et al. [28] classified the races of maize in Mexico based on their environmental conditions into four groups: temperate to semi-hot, semi-hot to hot, very hot, and a special group for the Jala race and a few associates. In a similar fashion, Hartkamp et al. [48] classified maize environments into three mean temperature conditions (mean annual temperature <18 C, ≥18 to <24, and ≥24) and Brush and Perales [30] found that the maize environments of Chiapas could be classified in three main groups (hot, semi-hot and temperate) based on altitude and the frequency of the races present. In Mexico, landraces are typically dominant in temperate to semi-hot environments and commercial cultivars and their advanced generations are common in hot or very-hot environments [30].

The 11 geographic regions we have proposed integrate the distributions for maize races and not all of these are adapted to the entire region. Greatest racial diversity is associated with regions with complex environments. Thus, the Sierra Madre Occidental and Sierra Madre del Sur have higher diversity than the uniform lowlands of the Yucatan Peninsula or the Gulf Plains. From the climate point of view of most of the maize regions we have defined have only one or two of the macro-environments defined above, except for the racially diverse Western Mountain Coastal Range, Valleys and Sierras of Oaxaca and Chiapas Complex that have three macro-environments. The exception to this generalization is the Eastern Sierra Madre in northeastern Mexico, which has a lower racial diversity than what might be expected for its environmental conditions, possibly reflecting its relatively recent human occupation compared to south and central Mexico. It also seems that temperate environments foment more diversity than hot environments; Ruiz et al. [28] characterized more races for temperate and semi-hot environments (15 and 14 races, respectively) than for very hot environments (9 races).

The delimitation of the biogeographic regions for maize coincided in a broad sense with a general consensus biogeography for Mexico, although there are some discrepancies mostly due to a smaller number of regions for maize. Biogeographic proposals have been made for Mexico based on several floristic or zoological groups, morpho-tectonic criteria, and a consensus scheme that has tried to integrate several of the specific proposals [49], [50]. The later, referred below as “consensus biogeography”, was proposed by a group of scientists in a workshop and has been extensively used in Mexico [49], [50]. Most of the regions defined here for maize correspond in their delimitation to two or more regions in the consensus biogeography of Mexico, others split a biogeographic region in a distinct way. For example, the Yucatan Peninsula region for maize corresponds to two regions of the consensus biogeography and Northern Plateau to four, and the Neovolcanic Axis of the consensus biogeography corresponds mostly to the Western Mountain Coastal Range although its eastern side is separated in the Central Plateau. The most outstanding differences are the Valleys and Sierras of Oaxaca and the Chiapas Complex regions in our maize biogeography, which contain complex environments and several regions of the consensus biogeography that do not correspond even roughly in delimitation. A particular difference is the Chihuahuan Canyons region for maize that is a specific area separated from the Sierra Madre Occidental of the consensus biogeography, which seems to closely match local ethnic populations. In these three cases there is no apparent correspondence with climatic or morphological factors or with any of the other biogeographic proposals. The maize models presented here relied solely on climate and maize collection variables and, thus, we can expect that the biogeographic regions we have defined might only reflect the underlying environment structure. However, this does not seem to be the case. The above suggests that even if the underlying structure of the biogeographic regions proposed for maize are in large part determined by climatic or physiographic factors, other drivers are being expressed.

It has long been proposed that maize diversity is associated with the distribution of the indigenous peoples of Mexico [1], [27], [45], [51]. Mexico has more than 60 ethnic groups and most of those living in rural areas have cultivated traditional varieties of maize uninterruptedly for hundreds of years. Nonetheless, associations between maize richness or maize biogeographic regions with ethnic groups are not straightforward. We found several significant correlations between presence of ethnic populations (as measured by number of ethnic groups or total population of ethnic groups) and race richness for several biogeographic regions, although it should be noted that total population also correlates with race richness. Thus some of these correlations could be spurious and should be interpreted with care. Two regions with large populations of indigenous peoples (the Valleys and Sierras of Oaxaca and the Chiapas Complex) also have greater richness of maize races and endemic types (Table 1 and S2 Table). In the case of the Valleys and Sierras of Oaxaca the correlation is significant, but not so for the Chiapas Complex. Two other regions have also relatively large populations of indigenous people but small richness of maize races (Gulf and Isthmus Plains and the Yucatan Peninsula), notwithstanding the correlations are significant, possibly because indigenous people are ubiquitous in the countryside of these regions. The Western Mountain Costal Range, one of the most diverse regions in number of maize races has a relatively small population of indigenous people, as is the case of the Northwestern Sierras. For these cases the correlation is significant and noteworthy because of the very small ethnic population. The Chihuahuan Canyons region is an interesting case because it seems to correspond to the area inhabited by the Rarámuri (Tarahumara), Tepehuan and Pima. However, upon closer inspection the area with highest diversity in that region is to the north of the area of inhabited by these ethnic groups (S4 Figure) and the correlation between indigenous population and race richness for this region is negative (though non-significant, S2 Table) and positive and significant for number of ethnic groups and richness. This case may be related to a history of displacement of ethnic populations to marginal environments, as has been the case in the Tarahumara region [52]. In any case, this ambivalent correlations in the Chihuahuan Canyons suggests a hypothesis to be tested. In a similar manner, areas within a biogeographic region for maize that have large ethnic populations do not coincide with highest diversity areas. This is the case of the Huichol, Tepehuano and Cora areas and of the Purepecha region in the Western Mountain Costal Range, and the Totonaca and Huastec in the Gulf and Isthmus Plains region. As mentioned above, the extensive Northern Plateau region is environmentally complex and could have higher maize diversity, but then sparse pre-Columbian populations and relatively recent population history has resulted in a smaller number of maize races. Richness seems to be determined mostly by climate, nonetheless the signature of human population history is also apparent [30]. Our biogeographic regions for maize can be seen as biocultural delimitations [53], even though cultural variables were not utilized for our models.

### Biogeographic regions, diversity and *in situ* conservation of maize

This determination of diversity regions and biogeographic regions can be used to guide efforts to conserve maize genetic resources. Greatest efforts should be focused in western Mexico and, in particular, there should be conservation efforts for races that are better represented in each biogeographic region. The high heterogeneity in racial composition and the presence of private races in several biogeographic regions suggests that a national conservation strategy will need to address all these regions, as not even the west of Mexico contains all the races. For example, an area with low race diversity, such as the Gulf and Isthmus Plains, is nevertheless important conservation wise because of the prevalence of Tuxpeño [13], an extremely important race in crop improvement, and the Northern Plateau also has low race diversity but is home to three races that are endemic to the region.

### Conclusions

Based on racial classification, diversity of maize in Mexico is greater in the west and south of the country. With the exception of the Yucatan Peninsula the areas with the least diversity had scarce or nomad populations before the 16th century. Based on racial composition Mexico can be divided into 11 biogeographic regions. Six of these regions are also centers of diversity and taken together contain more than 90% of the collections of 38 of the 47 races studied. These six centers coincide with Kato's four diversification regions for maize in Mexico, although two centers need to be divided because of distinct racial composition. Comparing overall race richness models for 1950, 1975 and 2005 indicate that major decline in diversity has not taken place, even though two regions suggest a possible decline in the 2005 models.

## Materials and Methods

Models for presence or absence were created for each race. The data base [54] was updated for the last time on September 2010 and has 22,931 accessions with 73 racial names of maize for collections between 1934 and 2010. More than 200 collectors from over 30 Mexican universities and institutions have been involved in assembling the sample. Criteria for collecting have varied, a common procedure has been gathering information on local variants in a community and then proceeding to pursue samples of 20 to 50 ears for each of these [38]. Some accessions (4,583) did not have minimum passport data (geo-referenced position and racial classification) and were discarded. We followed Ron et al. [31] for names of maize races; they list 59 labels of which two have been revised as synonyms [55]. Two races, Elotes Occidentales and Harinoso de Ocho, were reclassified as Bofo because these are difficult to distinguish and commonly confused, though Harinoso de Ocho had only 2 samples in the dataset. Eight recently proposed but not described races and two races not recognized as Mexican were eliminated. Additionally, 10 races that had been formally described but had less than 12 collections were also excluded in models by collection effort because of small sample size, though these were also modeled for the whole dataset. After these amendments 18,348 accessions and 47 racial names were kept (S1 Figure). The collections were done by more than 200 collectors in 32 of 33 States in Mexico (including the Federal District) and were taken from more than 1400 municipalities and more than 6000 communities. Racial classification was mostly done by the collectors but some samples were verified by the Gene Bank Curator at INIFAP (National Institute for Forestry, Agricultural and Animal Research) and other maize experts, in all 75 researchers determined the racial classification of the samples.

Generalized Additive Models [56] were created in R [57] for each race using climatic data available in WorldClim [58] with a resolution of 5 arc minutes (about 8.7×8.7 km or 75.7 km^2^ in central Mexico). Seven derived uncorrelated climate variables were used in the models based on previous principal component analysis of WorldClim data [59], and spatial trend variables (latitude and longitude) were included. Climate variables used [58] were maximum temperature in June, minimum temperature in January, difference between maximum and minimum monthly average, maximum difference between maximum and minimum daily temperature, rainfall in January, rainfall in June, and number of months with rainfall greater than 100 mm. Input to each model consisted of recorded presence points and 500 pseudo-absences drawn at random from the entire geographical region. The model methodology was based on finding a set of additive functions of climatic variables that led to the best discrimination between presence points and background points. Model of the binomial family were fit and the degree of complexity of the splines adjusted using internal cross validation. GAM models were constrained to use no more than 3 knots for climatic variables in order to ensure that responses had a unimodal form. Spatial trends were unconstrained. Model evaluation was based on the area under the receiver operator curve (AUC). AUC values were calculated for all models with more than 30 presence points by holding back 25% of the points for validation. AUC values were consistently above 0.8, demonstrating good discrimination. Model output was converted to range maps based on the receiver operator curve. A sensitivity threshold of 0.8 was used to avoid over-prediction that could have arisen through the inclusion of extreme distribution points. We combined the individual model outputs in order to obtain overall richness (number of races that occur within a grid cell).

The whole data base was analyzed for each race and also segmented for collection effort. Three intensive collection efforts were delimited by inspection of the data, collection effort 1950 = 1943–1954 (n = 1878), collection effort 1975 = 1968–1979 (n = 3568), and collection effort 2005 = 1997–2010 (n = 11,151, of which 6,666 of these were collected between 2007 and 2010). Only 485 accessions that had information for collection date were excluded in the segmented analysis. This procedure allowed the comparison of three independent models and the model for the entire database, which allowed informal corroboration of the distribution models. In general, models by collection effort were consistent between them and with the model for the whole data base. The models were inconsistent between collection effort for only three races and in four cases the general distribution model did not coincide with the models by collection effort.

Spatial analysis was done with Biodiverse software [60]. Distribution models were recreated for a 0.5×0.5 degree grid (36 grid cells of the distribution models) for models of 2005 collection effort. We used 2005 collection effort because it correlated best with the whole dataset and expresses present distributions. Race richness, endemism (central and whole), and biogeographic groups were done with a 8 grid neighborhood, clusters were formed with Sorenson index used as a dissimilarity measure [60]. Other measures of diversity were also calculated (beta diversity, Simpson, Shannon) but did not produce results of interest and are not presented.

## Supporting Information

S1 Figure **Collection points for all geo-referenced and classified accessions (n = 18,348).**
(TIF)

S2 Figure **Race richness (number of races occurring within each grid cell) determined by overlapping distribution models, for three collection efforts analyzed and the complete dataset.** a) 1950, b) 1975, c) 2005, and d) complete dataset. The maximum number of races is similar between models, suggesting no apparent reduction in diversity in more than 60 years. Interpretation of differences between models should consider sampling effects, approximate limits for diversity centers were drawn based on the overlap of the three collection efforts and the complete dataset models.
(ZIP)

S3 Figure **Biogeographic regions (clusters) produced by Biodiverse** [60] **spatial analysis.** These regions were redrawn based on physiographic subprovinces of INEGI [61], see Figure 1 in main text.
(TIF)

S4 Figure **Diversity areas for maize races and indigenous populations in Mexico.** a) race richness for the complete dataset, b) communities with 20% or more indigenous population.
(TIF)

S1 Table **Number of samples for maize races by collection effort.**
(DOCX)

S2 Table **Correlations between population of ethnic groups, total population and maize race richness for 2005 collection effort by biogeographic regions.**
(DOCX)

S1 Data **Dataset of georeferenced and classified collections used in models.**
(XLSX)

S2 Data **Definition of variables contained in dataset.**
(DOC)
