# Supplementary figures and images for: Mapping the Diversity of Maize Races in Mexico
Source: PLoS One. 2014 Dec 8;9(12):e114657. doi: 10.1371/journal.pone.0114657 (PMC4259470; doi:10.1371/journal.pone.0114657)

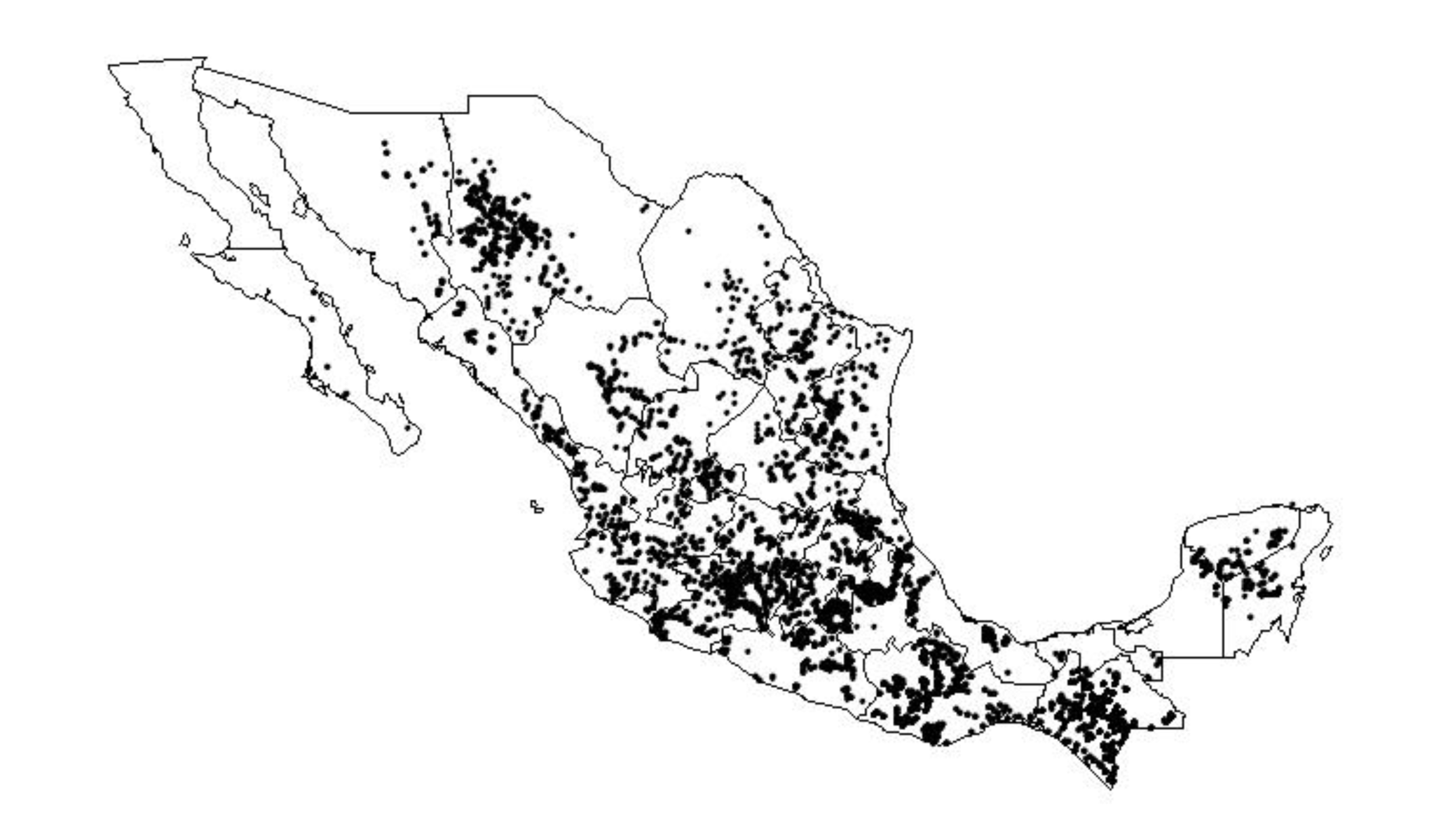

Supplement: S1 Figure — Collection points for all geo-referenced and classified accessions (n = 18,348). (TIF) [file pone.0114657.s001.tif]

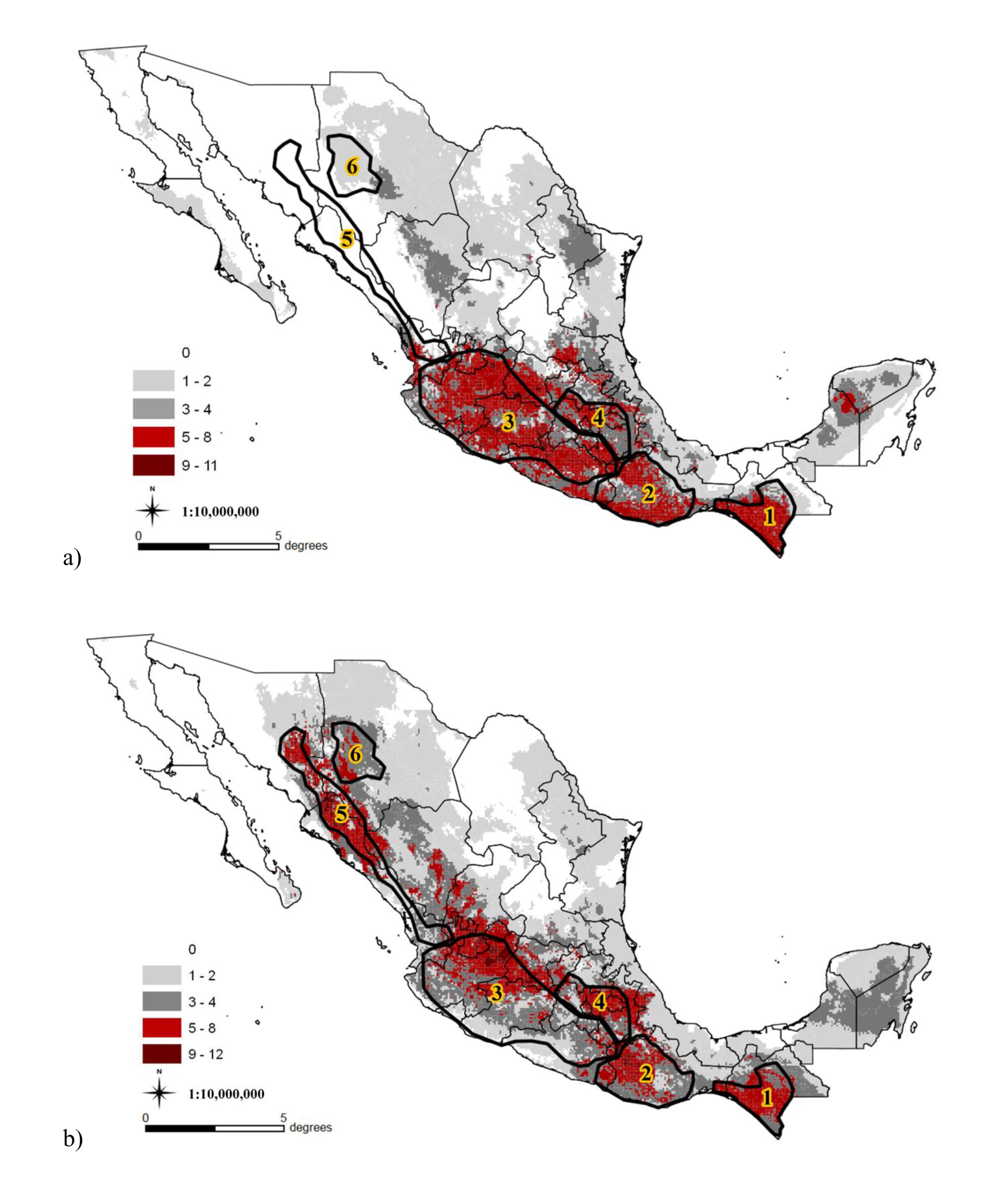

Supplement: S2 Figure — Race richness (number of races occurring within each grid cell) determined by overlapping distribution models, for three collection efforts analyzed and the complete dataset. a) 1950, b) 1975, c) 2005, and d) complete dataset. The maximum number of races is similar between models, suggesting no apparent reduction in diversity in more than 60 years. Interpretation of differences between models should consider sampling effects, approximate limits for diversity centers were drawn based on the overlap of the three collection efforts and the complete dataset models. (ZIP) [file pone.0114657.s002.zip › Figure S2_part 1.tif]

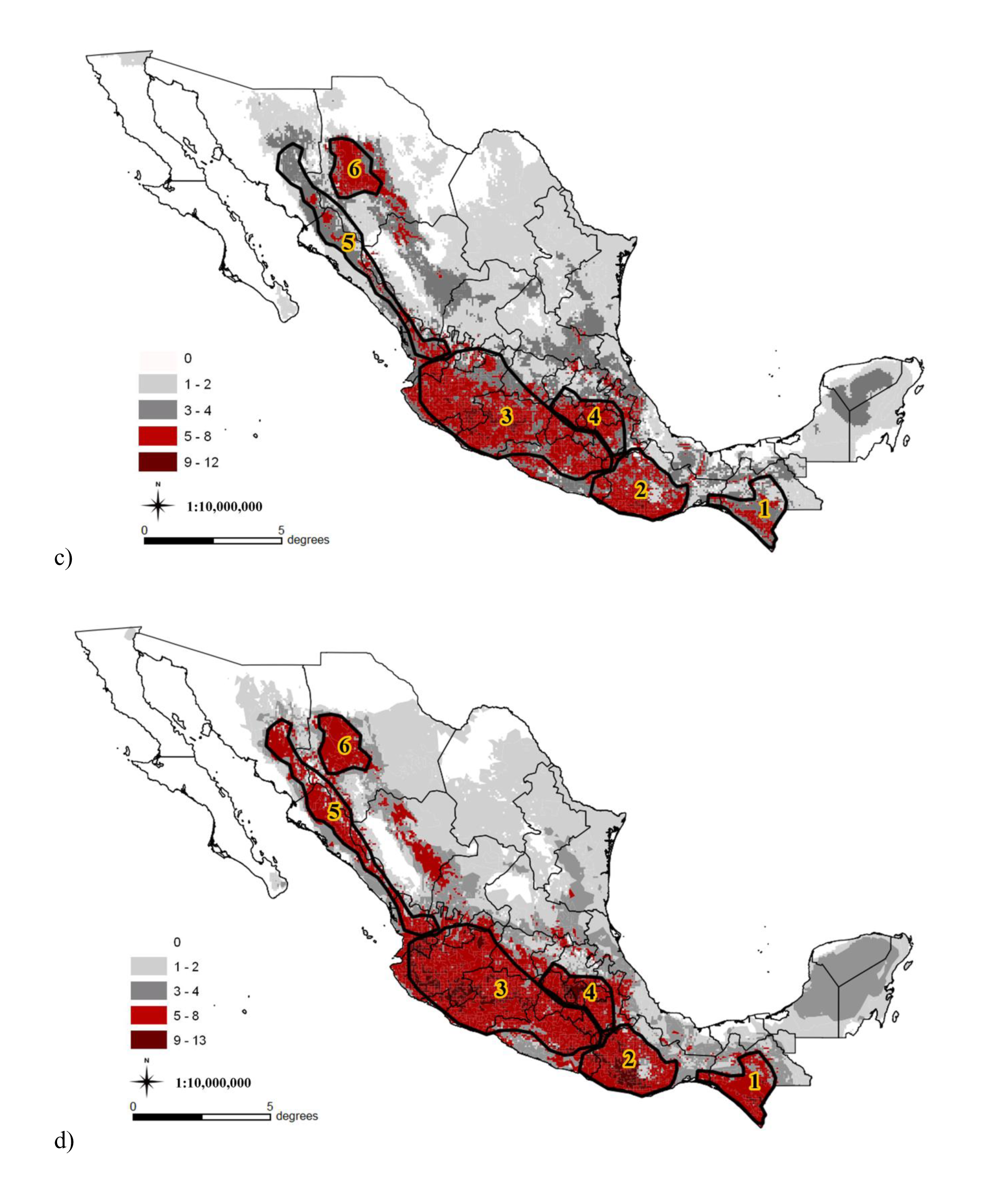

Supplement: S2 Figure — Race richness (number of races occurring within each grid cell) determined by overlapping distribution models, for three collection efforts analyzed and the complete dataset. a) 1950, b) 1975, c) 2005, and d) complete dataset. The maximum number of races is similar between models, suggesting no apparent reduction in diversity in more than 60 years. Interpretation of differences between models should consider sampling effects, approximate limits for diversity centers were drawn based on the overlap of the three collection efforts and the complete dataset models. (ZIP) [file pone.0114657.s002.zip › Figure S2_part 2.tif]

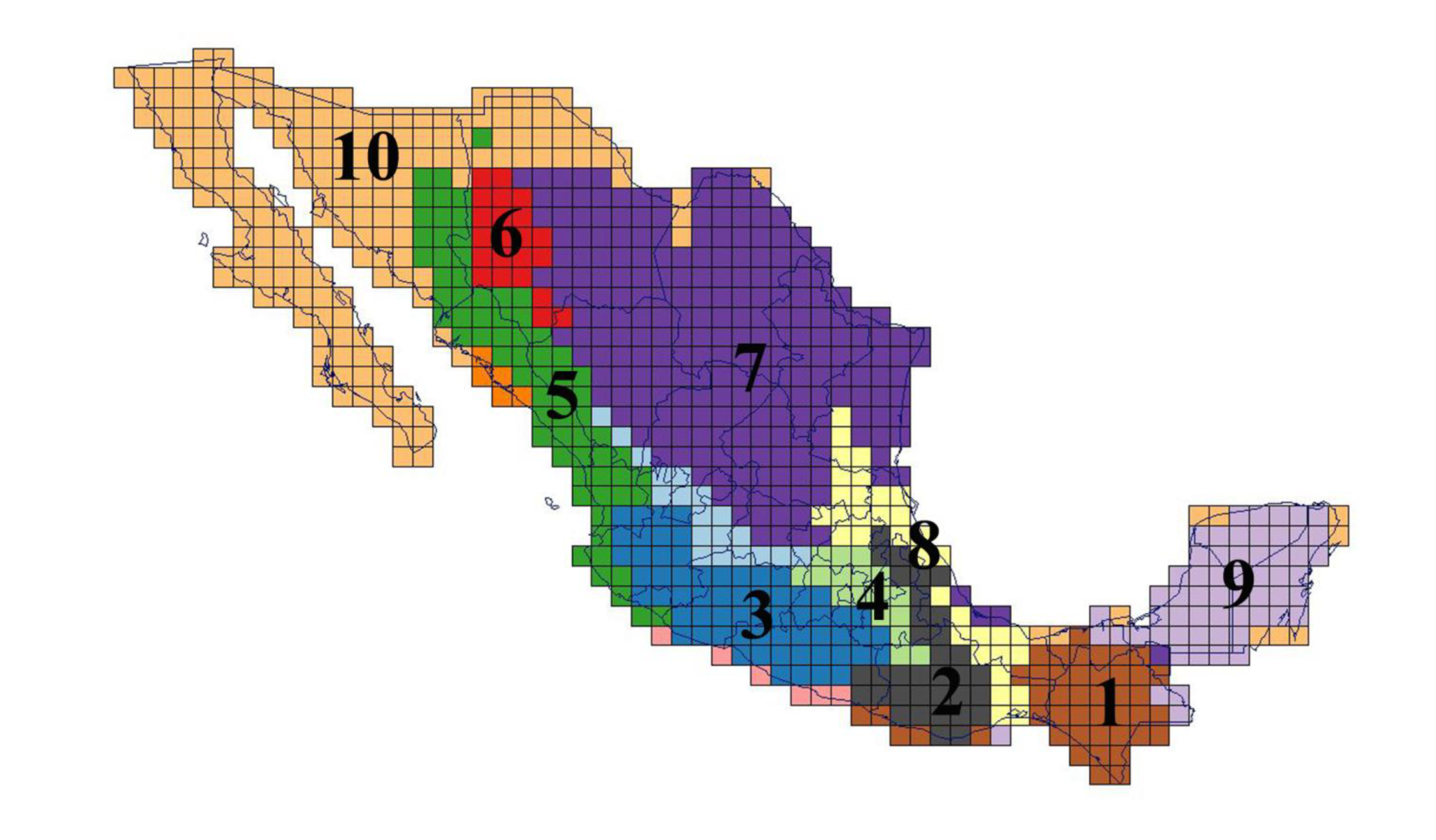

Supplement: S3 Figure — Biogeographic regions (clusters) produced by Biodiverse [60] spatial analysis. These regions were redrawn based on physiographic subprovinces of INEGI [61], see Figure 1 in main text. (TIF) [file pone.0114657.s003.tif]

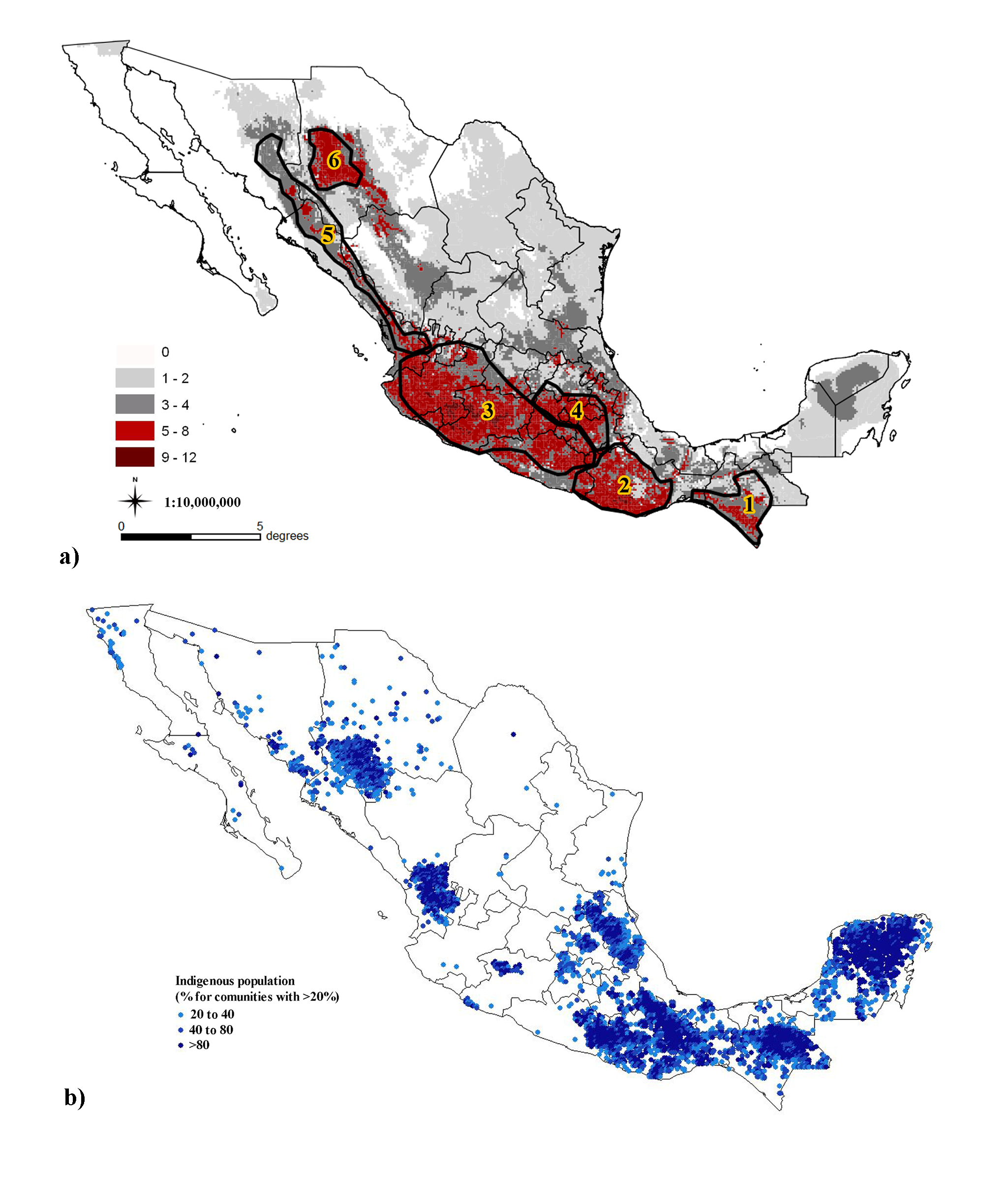

Supplement: S4 Figure — Diversity areas for maize races and indigenous populations in Mexico. a) race richness for the complete dataset, b) communities with 20% or more indigenous population. (TIF) [file pone.0114657.s004.tif]
