# Supplementary material for: Mapping the Diversity of Maize Races in Mexico
Source: PLoS One. 2014 Dec 8;9(12):e114657. doi: 10.1371/journal.pone.0114657 (PMC4259470; doi:10.1371/journal.pone.0114657)
Supplement: S1 Table — Number of samples for maize races by collection effort. (DOCX) [file pone.0114657.s005.docx]

Table S1. Number of samples for maize races by collection effort.

| Race | 1950 (1943-1954) | 1975 (1968-1979) | 2005 (1997-2010) | Not dated | Total |
| --- | --- | --- | --- | --- | --- |
| **Races included in models** |  |  |  |  |  |
| Ancho | 4 | 45 | 245 | 27 | 321 |
| Apachito | 0 | 19 | 46 | 0 | 65 |
| Arrocillo Amarillo | 34 | 156 | 108 | 13 | 311 |
| Azul | 1 | 6 | 85 | 1 | 93 |
| Bofo | 36 | 55 | 326 | 11 | 428 |
| Bolita | 47 | 117 | 349 | 17 | 530 |
| Cacahuacintle | 9 | 14 | 32 | 9 | 64 |
| Celaya | 180 | 319 | 269 | 137 | 905 |
| Chalqueño | 80 | 169 | 181 | 180 | 610 |
| Chapalote | 2 | 18 | 10 | 0 | 30 |
| Comiteco | 48 | 74 | 1115 | 42 | 1279 |
| Complejo Serrano de Jalisco | 13 | 0 | 2 | 3 | 18 |
| Conejo | 32 | 8 | 65 | 6 | 111 |
| Cónico | 195 | 589 | 641 | 521 | 1946 |
| Cónico Norteño | 193 | 277 | 570 | 125 | 1165 |
| Coscomatepec | 9 | 67 | 42 | 0 | 118 |
| Cristalino de Chihuahua | 9 | 99 | 197 | 4 | 309 |
| Dulcillo del Noroeste | 2 | 26 | 4 | 3 | 35 |
| Dzit Bacal | 30 | 39 | 34 | 5 | 108 |
| Elotero de Sinaloa | 2 | 1 | 70 | 1 | 74 |
| Elotes Cónicos | 40 | 104 | 334 | 85 | 563 |
| Gordo | 2 | 12 | 55 | 0 | 69 |
| Jala | 23 | 10 | 7 | 0 | 40 |
| Maíz Blando de Sonora | 1 | 23 | 15 | 3 | 42 |
| Maíz Dulce | 8 | 12 | 33 | 3 | 56 |
| Mushito | 32 | 43 | 324 | 72 | 471 |
| Nal-Tel | 39 | 51 | 41 | 6 | 137 |
| Nal-Tel de Altura | 7 | 8 | 46 | 1 | 62 |
| Olotillo | 73 | 72 | 939 | 8 | 1092 |
| Olotón | 11 | 59 | 513 | 8 | 591 |
| Onaveño | 9 | 30 | 34 | 12 | 85 |
| Palomero Toluqueño | 20 | 9 | 11 | 10 | 50 |
| Pepitilla | 26 | 40 | 167 | 70 | 303 |
| Ratón | 29 | 62 | 313 | 35 | 439 |
| Reventador | 16 | 32 | 37 | 0 | 85 |
| Serrano Mixe | 0 | 0 | 37 | 2 | 39 |
| Tablilla de Ocho | 7 | 14 | 13 | 8 | 42 |
| Tabloncillo | 101 | 72 | 336 | 87 | 596 |
| Tabloncillo Perla | 45 | 90 | 49 | 14 | 198 |
| Tehua | 8 | 4 | 31 | 0 | 43 |
| Tepecintle | 23 | 62 | 420 | 9 | 514 |
| Tuxpeño | 247 | 516 | 2489 | 149 | 3401 |
| Tuxpeño Norteño | 87 | 38 | 148 | 31 | 304 |
| Vandeño | 47 | 31 | 215 | 15 | 308 |
| Zamorano Amarillo | 16 | 17 | 29 | 15 | 77 |
| Zapalote Chico | 18 | 38 | 79 | 3 | 138 |
| Zapalote Grande | 17 | 21 | 45 | 0 | 83 |
| Total samples used in models | 1878 | 3568 | 11,151 | 1751 | 18,348 |
|  |  |  |  |  |  |
| **Described races not included in models** |  |  |  |  |  |
| Chatino Maizón | 0 | 0 | 0 | 0 | 0 |
| Choapaneco | 0 | 0 | 0 | 5 | 5 |
| Harinoso de Ocho | 1 | 0 | 1 | 2 | 4 |
| Mixeño | 0 | 10 | 0 | 11 | 21 |
| Mixteco | 0 | 0 | 0 | 4 | 4 |
| Motozinteco | 0 | 4 | 0 | 5 | 9 |
| Mountain Yellow | 4 | 0 | 7 | 5 | 16 |
| Negrito | 2 | 0 | 6 | 8 | 16 |
| Negro de Chimaltenango | 1 | 1 | 0 | 2 | 4 |
| Palomero de Chihuahua | 0 | 4 | 2 | 6 | 12 |
| Palomero de Jalisco | 3 | 0 | 0 | 3 | 6 |
|  |  |  |  |  |  |
| **Undescribed races not included in models** |  |  |  |  |  |
| Amarillo de Tierra Caliente raza nov. | 0 | 0 | 5 | 0 | 5 |
| Argentino | 0 | 0 | 7 | 0 | 7 |
| Cubano Amarillo | 1 | 11 | 53 | 0 | 65 |
| Maíz de Ecuaro raza nov. | 0 | 0 | 58 | 0 | 58 |
| Maizón raza nov. | 0 | 0 | 18 | 0 | 18 |
| Prieto de Tierra Caliente raza nov. | 0 | 0 | 25 | 0 | 25 |
| Purhépecha raza nov. | 0 | 0 | 22 | 0 | 22 |
| Quicheño | 0 | 1 | 0 | 1 | 1 |
| Serrano | 0 | 0 | 8 | 0 | 8 |
| Tamaulipas raza nov. | 0 | 0 | 58 | 0 | 58 |
| Tsïri Charhápiti raza nov. | 0 | 0 | 29 | 0 | 29 |
| Uruapeño | 0 | 0 | 0 | 1 | 1 |
