# Supplementary material for: Mapping the Diversity of Maize Races in Mexico
Source: PLoS One. 2014 Dec 8;9(12):e114657. doi: 10.1371/journal.pone.0114657 (PMC4259470; doi:10.1371/journal.pone.0114657)
Supplement: S2 Table — Correlations between population of ethnic groups, total population and maize race richness for 2005 collection effort by biogeographic regions. (DOCX) [file pone.0114657.s006.docx]

Table S2. Correlations between population of ethnic groups, total population and maize race richness for 2005 collection effort by biogeographic regions.

| Biogeographic region | n | Mayor ethnic groups | Number of ethnic groups | Ethnic population | | Race richness (mean) | Spearman rho | |
| --- | --- | --- | --- | --- | --- | --- | --- | --- |
|  |  |  |  | 10^6^ | % of total population |  | Number of ethnic groups | Ethnic population |
| Chiapas Complex | 42 | Tzotzil, Tzeltal, Tojolabal, Zoque, Chol | 20 | 1.256 | 18.2 | 5.4 | 0.20 | 0.13 |
| Oaxacan Valleys and Sierras | 38 | Mixtec, Mazatec, Zapotec, Chinantec, Popoloca, Nahuatl | 18 | 2.085 | 25.9 | 11.5 | 0.24+ | 0.46** |
| Western Costal Mountain Range | 69 | Amuzgo, Triqui, Purepecha, Nahuatl, Tepehua, Cora, Huichol | 5 | 0.484 | 2.7 | 11.1 | 0.25* | 0.14 |
| Central Plateau | 17 | Nahuatl, Mazahua, Matlatzinteca, Otomi | 9 | 0.677 | 2.4 | 9.1 | 0.20 | 0.20 |
| Northwest Sierras | 69 | Yaqui, Mayo | 9 | 0.128 | 3.0 | 4.7 | 0.15 | 0.33** |
| Chihuahuan Canyons | 23 | Tarahumara, Tepehuan, Pima | 5 | 0.054 | 13.0 | 7.0 | 0.45** | -0.18 |
| Northern Plateau | 201 | Kickapoo, Alto Seminole Creole | 8 | 0.131 | 5.7 | 2.7 | 0.39** | 0.32** |
| Gulf and Isthmus Plains | 40 | Totonac, Tepehua, Popoluca, Zoque, Zapotec, Huave, Huastec, Pame, Nahuatl | 17 | 1.045 | 13.8 | 4.8 | 0.41** | 0.31+ |
| Yucatan Peninsula | 63 | Yucatec Maya | 8 | 0.851 | 19.9 | 1.8 | 0.62** | 0.48** |
| Bajio | 19 | Chichimeca, Nahuatl, Otomi | 2 | 0.321 | 1.0 | 7.4 | 0.32 | 0.18 |
| Baja California and Northwest | 123 | Mixtec (immigrants) | 10 | 0.105 | 1.6 | 0.4 | 0.19** | 0.03 |
| Mean for Mexico | 733 |  |  | 6.867 | 6.1 | 4.0 | 0.46** | 0.43** |

Notes: Ethnic and total population from INEGI (Instituto Nacional de Estadística y Geografía, Censo de Población y Vivienda 2010, available at: http://www.inegi.org.mx). Variables calculated for 0.5 degree grid cells; richness is not identical to Table 2 because of larger grid size, n is for number of cells in correlations. Significance levels for correlations: +=0.10, *=0.05, **=0.01.
