# Supplementary material for: Mapping the Diversity of Maize Races in Mexico
Source: PLoS One. 2014 Dec 8;9(12):e114657. doi: 10.1371/journal.pone.0114657 (PMC4259470; doi:10.1371/journal.pone.0114657)
Supplement: S2 Data — Definition of variables contained in dataset. (DOC) [file pone.0114657.s008.doc]

GLOBAL MAIZE PROJECT DATASET

The dataset (last updated in December 2010) has integrated 21,993 georeferenced records of maize collections from 34 specific datasets organized by different institutions and national researchers and sponsored under the project entitled “*Recopilación, generación, actualización y análisis de información acerca de la diversidad genética de maíces y sus parientes silvestres en México*” (Collection, generation, update and analysis of information about the genetic diversity of maize and its wild relatives) coordinated by CONABIO (National Commission for the Cognizance and Use of Biodiversity) in association with INE (National Institute of Ecology, of the Secretariat of Environment and Natural Resources) and INIFAP (National Institute for Forestry, Agricultural and Livestock Research). In 18,348 accessions maize is classified for race type. The complete dataset is available at [http://www.biodiversidad.gob.mx/genes/pdf/proyecto/Anexo13_Base%20de%20datos/BaseMaicesNativos.xlsx](http://www.biodiversidad.gob.mx/genes/pdf/proyecto/Anexo13_Base de datos/BaseMaicesNativos.xlsx). A partial dataset is associated with this file and contains the variables used in the article “Mapping the diversity of maize races in Mexico”

Variable names and definitions:

Id

Identifier, unique and consecutive for all dataset.

NumeroDeCatalogo

Identifier for sample with which the accession was registered in the particular collection of the dataset.

AnioColecta

Year of collection.

ApellidoPaterno(Colector)

Father surname of collector.

ApellidoMaterno(Colector)

Mother surname of collector.

Nombre(Colector)

Given name of collector.

Raza primaria

Primary race associated with collection.

Influencia de otras razas

Influence of other races associate with collection as described by classifier.

ApellidoPaterno(Determinador)

Father surname of whom determined the race type.

ApellidoMaterno(Determinador)

Mother surname of whom determined the race type.

Nombre(Determinador)

Given name of whom determined the race type.

Estado

State of collection.

Municipio

Municipality of collection.

Localidad

Locality of collection.

Altitud

Altitud of collection.

Longitud

Longitude of collection in decimal degrees.

Latitud

Latitude of collection in decimal degrees.
